# Interaction Between Leg Muscle Performance and Sprint Acceleration Kinematics

**DOI:** 10.1515/hukin-2015-0109

**Published:** 2015-12-30

**Authors:** Robert G. Lockie, Farzad Jalilvand, Samuel J. Callaghan, Matthew D. Jeffriess, Aron J. Murphy

**Affiliations:** 1 Department of Kinesiology, California State University, Northridge, Northridge, USA; 2 School of Exercise and Health Sciences, Edith Cowan University, Joondalup, Australia; 3 Faculty of Health, University of Technology, Sydney, Lindfield, Australia; 4 Sports Studies, Exercise and Sports Science, and Clinical Exercise Physiology Department, School of Science and Technology, University of New England, Armidale, Australia

**Keywords:** step kinematics, leg power, musculotendinous stiffness, squat strength, field sports

## Abstract

This study investigated relationships between 10 m sprint acceleration, step kinematics (step length and frequency, contact and flight time), and leg muscle performance (power, stiffness, strength). Twenty-eight field sport athletes completed 10 m sprints that were timed and filmed. Velocity and step kinematics were measured for the 0–5, 5–10, and 0–10 m intervals to assess acceleration. Leg power was measured via countermovement jumps (CMJ), a five-bound test (5BT), and the reactive strength index (RSI) defined by 40 cm drop jumps. Leg stiffness was measured by bilateral and unilateral hopping. A three-repetition maximum squat determined strength. Pearson’s correlations and stepwise regression (p ≤ 0.05) determined velocity, step kinematics, and leg muscle performance relationships. CMJ height correlated with and predicted velocity in all intervals (r = 0.40–0.54). The 5BT (5–10 and 0–10 m intervals) and RSI (5–10 m interval) also related to velocity (r = 0.37–0.47). Leg stiffness did not correlate with acceleration kinematics. Greater leg strength related to and predicted lower 0–5 m flight times (r = −0.46 to −0.51), and a longer 0–10 m step length (r = 0.38). Although results supported research emphasizing the value of leg power and strength for acceleration, the correlations and predictive relationships (r^2^ = 0.14–0.29) tended to be low, which highlights the complex interaction between sprint technique and leg muscle performance. Nonetheless, given the established relationships between speed, leg power and strength, strength and conditioning coaches should ensure these qualities are expressed during acceleration in field sport athletes.

## Introduction

Sprinting speed is an essential component of field-based sports, such as soccer, rugby, and Australian and American football. An important consideration for field sport athletes is that during match-play, maximal speed is generally not attainable, as in-game sprints tend to be relatively short. For example, maximal sprints in soccer (Bangsbo et al., 1991) and Australian football (Dawson et al., 2004) are often 10 m or less. This emphasizes the importance of acceleration ability for field sport athletes. Sprint acceleration involves the interaction of many physical characteristics, including technique, leg power, musculotendinous stiffness and strength (Hunter et al., 2004; Lockie et al., 2011; Nimphius et al., 2010). Understanding the interaction between these factors is critical for the strength and conditioning coach.

Sprinting is often described by step kinematics. Step length is the distance between alternating contacts of each foot; step frequency is the rate at which steps can be reproduced. Contact time is the duration when a leg is in ground support; flight time is the period when the athlete is airborne. Previous research has illustrated the importance of both a high step length (Callaghan et al., 2014; Lockie et al., 2013) and frequency (Lockie et al., 2011; Murphy et al., 2003) for acceleration. Lower contact times have also been linked to efficient acceleration (Lockie et al., 2011; Murphy et al., 2003). Sayers (2000) recommended shorter flight times for athletes involved in contact sports such as rugby union, American and Australian football, as players cannot change direction once airborne, and this can affect their body position prior to a collision. Due to the importance of these characteristics, it is valuable to understand how physical qualities may influence them.

Relationships between leg power (Cronin and Hansen, 2005; Lockie et al., 2011), stiffness (Bret et al., 2002; Lockie et al., 2011), strength (Cronin and Hansen, 2005; Lockie et al., 2011; Spiteri et al., 2013), and speed have been investigated. Leg power is often measured indirectly through jump testing (Lockie et al., 2014); one such measure is the countermovement jump (CMJ). The CMJ has previously been shown to correlate to speed over 10 m in rugby league players (Cronin and Hansen, 2005), and recreational field sport athletes (Lockie et al., 2011). Faster field sport athletes have also been found to have greater reactive power, as determined by the reactive strength index (RSI) from a 40 cm drop jump (Lockie et al., 2011). Musculotendinous leg stiffness relates to the ability to absorb, store and release energy imposed by the strain of an impact, and has been associated with speed increases from acceleration to maximum velocity during a 100 m sprint (Bret et al., 2002). Absolute strength as measured by a three-repetition maximum back squat (3RM) has shown a lack of correlation with speed in rugby league players (Baker and Nance, 1999; Cronin and Hansen, 2005) and field sport athletes (Lockie et al., 2011). However, Baker and Nance (1999) found that when the 3RM squat was made relative to body mass, a relationship to sprint performance was established. Nevertheless, no research has analyzed the extent to which leg muscle performance can influence acceleration step kinematics.

As a result, this study investigated the relationship between 10 m sprint performance and leg power (five-bound test [5BT], CMJ, RSI), stiffness (bilateral and unilateral hopping), and strength (3RM squat) in male field sport athletes. It was hypothesized that leg power, strength, and stiffness would correlate with and predict sprint velocity and step kinematics. This research provides beneficial information for strength and conditioning coaches, as it shows potential implications for the selected leg muscle performance tasks on the kinematics produced during acceleration.

## Material and Methods

### Participants

Twenty-eight male field sport athletes (age = 22.36 ± 2.83 years; body height = 1.80 ± 0.06 m; body mass = 82.42 ± 7.61 kg) were recruited. Participants were included in the study if they: were 18 years of age or older; were active in a field sport (rugby union, rugby league, Australian football, soccer); had a strength and field sport training history (≥three training sessions per week) extending over the previous year; and did not have any medical conditions that would compromise study participation. Although there may be some differences in traits between participants from different sports, the analysis of performance with regard to physical characteristics common to athletes from assorted team sports has been conducted in the literature, due to movement demand similarities between sports (i.e. all of these sports require sprint acceleration) (Lockie et al., 2011; Lockie et al., 2014; Sassi et al., 2009; Sekulic et al., 2013; Spiteri et al., 2013). Additionally, the inclusion criteria were implemented to ensure that all participants had a similar training background, regardless of the sports discipline they practiced. The research was approved by the University of Technology, Sydney ethics committee. All participants received a clear explanation of the study, including the risks and benefits of participation. Written informed consent was obtained prior to testing.

### Procedures

Testing was conducted over two days, separated by 48 hours. Day 1 consisted of 10 m sprint tests and the leg power assessments (5BT, CMJ, 40 cm drop jumps). Bilateral and unilateral hopping leg stiffness tests and the 3RM squat were performed on day 2. Prior to data collection on day 1, the participant’s age, body height and mass were recorded. A standardized warm-up, consisting of 10 min of self-paced jogging, 10 min of dynamic stretching of the lower limbs, and progressive speed runs, was used for all participants. They were tested at the same time of day for both sessions, and refrained from intensive exercise and stimulants in the 24 hours prior to testing.

### Sprint Kinematics Assessment

A 10 m sprint was used to assess acceleration kinematics (Kawamori et al., 2013; Lockie et al., 2011; Lockie et al., 2013; Nimphius et al., 2010). Time was measured by a velocimeter (Onspot, Wollongong, Australia), which consisted of a stopwatch (Seiko, Tokyo, Japan), and a nylon string attached to a reel. The string was attached to the back of the participant’s shorts and unwound during the sprint. The velocimeter was placed on a 0.72 m high table, 1.5 m behind the participant. The stopwatch was electronically triggered with the participant’s first movement. If the timer was falsely triggered, the trial was stopped and reattempted. Times were recorded for the 0–5 m, 5–10 m, and 0–10 m intervals. The times for the three intervals were used to calculate velocity through the equation: *velocity = displacement*·*time*^−^*^1^*.

A high-speed video camera (Peak Performance Technologies, Englewood, USA), with a sampling rate of 200 Hz, recorded the sprints for the step kinematics assessment. The camera was positioned 8.75 m lateral (perpendicular to the sprint direction) to the participant. Prior to testing, a standard meter ruler was carried throughout the observation volume and recorded to provide a calibration scale for video analysis (Gruen, 1997). This data was exported and analyzed within custom software (UTS Kinematic Data Collection Software, Lindfield, Australia) to ensure images were representative of the real-space coordinate system. The 0–5 m and 5–10 m intervals were filmed separately, which had been done in previous research (Lockie et al., 2012a; Lockie et al., 2013). Three trials each measured kinematics for the two intervals, for a total of six trials. Three minutes between-trial recovery was provided. For the 0–5 m and 5–10 m intervals, the camera was positioned at 2.5 m and 7.5 m perpendicular to the sprint direction, respectively. A television (Panasonic, Osaka, Japan) and video recorder (Peak Performance Technologies, Englewood, USA) were connected to the camera to record each trial. Two 500-Watt lights provided external illumination.

Reflective tape was placed on the head of the fifth metatarsal on the right foot, and head of the first metatarsal on the left foot, to allow step kinematics computation. Recordings were transferred onto a computer (Dell Inc., Round Rock, USA) and exported into the custom software for analysis. All trials were analyzed, and averages calculated. The total number of steps and contacts a participant had within an interval was used to determine mean step kinematics. Start and finish points of movement phases were visually determined from the video footage by using the frame-by-frame tool on the computer software to scroll through the recording (Bradshaw et al., 2007; Lockie et al., 2011). Contact time was the period between touchdown (first instance when the foot contacted the ground), and toe-off (first instance when the foot broke contact with the ground), of one foot during stance. Flight time was the duration between toe-off and touchdown of the opposing foot. Step length was the distance between toe-off of one foot, and touchdown of the opposing foot. Step frequency was calculated through the formula: *step frequency = velocity·mean step length per interval*^−^*^1^* (Hunter et al., 2004).

### Leg Power Assessment

For each power assessment, three trials were completed with 2 min of between-trial recovery, and averages were computed. The 5BT indirectly measured power in the horizontal plane. Participants covered the greatest distance possible by performing a series of five forward bounds with alternate right- and left-foot contacts. They started with both feet parallel, and chose the preferred leg to perform the initial push-off with, which was the same for each trial. Distance was measured from the start line to the final position of the front of the landing foot on the fifth bound (Lockie et al., 2011; Lockie et al., 2012a). Average 5BT distance was also made relative to body mass via the equation: *relative 5BT (m*·*kg*^−^*^1^) = 5BT distance*·*body mass*^−^*^1^*.

The CMJ provided an indirect measure of vertical power. A force plate (Onspot, Wollongong, Australia), sampling at 1000 Hz, recorded the trials. Data from the force plate was recorded to computer via a National Instruments (DAQCard™-AI-16E-4) analogue-to-digital converter. Participants stood on the plate and jumped for maximal height when directed. No restrictions were placed on countermovement range of motion, participants kept their hands on their hips throughout the jump to restrict upper-body contributions, and maintained straight legs whilst airborne. Flight time was recorded, and used to calculate jump height through the formula: *jump height =* ½·*acceleration due to gravity [9.8 m·s*^−^*^2^]*· *(flight time*·*2*^−^*^1^)^2^*. The use of projectile motion equations to calculate jump height has been used in the literature (Cronin and Hansen, 2005; Lockie et al., 2011). The power index from the average CMJ was also calculated by using the Lewis formula: *Power (kg·m·s*^−^*^1^) = √4.9·Body Mass·√vertical jump height* (Harman et al., 1991).

A 40 cm drop jump determined the RSI, which assesses the ability to produce force rapidly under high eccentric load. The 40 cm height was chosen as it had been recommended for power training (Bobbert et al., 1987). The drop jumps were performed on the force plate. The participants’ started by standing upright on a 40 cm box. They were instructed to keep their hands on their hips throughout the jump, step off from the height, and to jump maximally upon landing on the force plate, attempting to minimize contact time (duration of force plate contact following the drop). Contact time and the flight time of the jump were recorded. Jump height was calculated using the previously stated formula. The RSI was determined by the equation: *RSI = jump height*·*contact time*^−^*^1^* (Flanagan and Comyns, 2008; Lockie et al., 2011).

### Leg Stiffness Assessment

Vertical leg stiffness was determined using bilateral and unilateral hopping tests conducted on the force plate. Participants hopped at 2.2 Hz in time with a digital metronome (Seiko, Tokyo, Japan), which is the preferred frequency for hopping (Farley et al., 1991). They kept their hands on their hips throughout each trial to negate upper-body contribution. No shoes were worn during the hopping tests. Trials lasted for approximately 10 s, or until a steady hopping state was achieved. Feedback about technique was provided to aid the participant in achieving steady-state hopping. Once synchronization with the metronome was achieved, kinetic data from the force plate was collected for 5 s. Trials were accepted when hops were within 2% of the required movement frequency (Ferris and Farley, 1997). Three acceptable, consecutive hops were selected for analysis, and averages were used (McLachlan et al., 2006). Stiffness was calculated as the ratio of the maximum ground reaction force, and the maximum change in length of the ‘internal leg spring’ (the muscles of the multi-jointed leg) occurring from the start of ground contact, to the point where the leg muscles were maximally compressed at the ground reaction force peak: *leg stiffness = maximum ground reaction force*·*maximum leg spring change of displacement*^−^*^1^* (Ferris and Farley, 1997). Leg stiffness was made relative to body mass for the final analysis through the equation: *relative stiffness = stiffness*·*body mass*^−^*^1^*.

### Leg Strength Assessment

A 3RM back squat was used to assess lower-body strength (Baker and Nance, 1999; Cronin and Hansen, 2005; Lockie et al., 2011) and was conducted on a Smith machine (Life Fitness, Artarmon, Australia). The warm up consisted of 15 body weight squats, followed by 10 repetitions at 60–70% of the participants’ estimated maximum squat, which was based on their previous training experience. After a 3 min rest, participants completed their first attempt. The weight was increased until the participant failed to complete three repetitions. No more than five attempts were generally needed before the 3RM was reached. For the down phase, participants descended until the tops of their thighs were parallel to the floor. A length of non-weight-bearing wire, individually set for each participant, was tied across the Smith machine at the descent height to provide an indication of the required depth (Cotterman et al., 2005; Lockie et al., 2011). This was visually assessed by the researcher, and verbal cues were provided to participants on when to stop the down phase, and begin the up phase of the squat. If the participant did not descend appropriately, the trial was disregarded and reattempted after the 3 min rest period. Absolute strength was the maximum load lifted for three repetitions. Relative strength was derived through the equation: *relative strength = 3RM*·*body mass*^−^*^1^*.

### Statistical Analyses

Statistical analyses were computed using the Statistics Package for Social Sciences (Version 22.0; IBM Corporation, New York, USA). Descriptive statistics (mean ± standard deviation; 95% confidence intervals [CI]) were calculated for each variable. The Levene statistic determined data homogeneity of variance. Pearson’s correlation analysis (*p* ≤ 0.05) compared relationships between velocity and step kinematics, and leg power, stiffness, and strength. The correlation coefficient (r) strength was designated as per (Hopkins, 2009). An r value between 0 to 0.30, or 0 to −0.30, was considered small; 0.31 to 0.49, or −0.31 to −0.49, moderate; 0.50 to 0.69, or −0.50 to −0.69, large; 0.70 to 0.89, or −0.70 to −0.89, very large; and 0.90 to 1, or −0.90 to −1, near perfect for predicting relationships. Stepwise multiple regression analyses (*p* ≤ 0.05) were conducted for velocity, step length and frequency, and contact and flight times, for all intervals (each was a dependent variable), with the leg power, stiffness, and strength.

## Results

**Figure 1:**
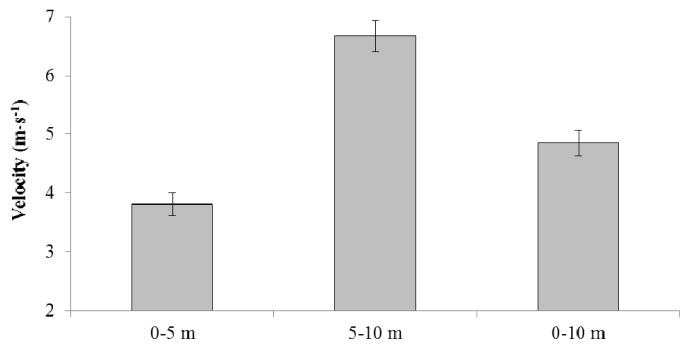
0–5 m, 5–10 m, and 0–10 m velocity for a 10 m sprint in experienced male field sport athletes (n = 28)

Figure 1 displays the velocity for each interval; step kinematics, leg power, stiffness, and strength data are shown in Table 1. The correlations between sprint velocity and step kinematics, and the leg muscle performance tests, are presented in Table 2. CMJ height positively correlated with velocity in each interval. 0–5 m (p = 0.04) and 0–10 m (p = 0.01) correlations were moderate; the 5–10 m (p < 0.01) correlation was large. The 5BT moderately correlated with 5–10 m (p = 0.01) and 0–10 m (p = 0.04) velocity. The relative 5BT (p = 0.02) and the RSI (p = 0.05) moderately correlated with 5–10 m velocity. There were three significant correlations between step kinematics and the leg muscle performance tests. Absolute 3RM squat strength negatively related to 0–5 m flight time (p = 0.01). Relative 3RM strength negatively related to 0–5 m flight time (p = 0.01), and positively related to 0–10 m step length (p = 0.05).

Table 3 displays the regression data for velocity and selected step kinematics. For velocity in each interval, CMJ height was the best predictor. The strength of these relationships tended to be low. Only two step kinematic variables demonstrated significant predictive relationships. 0–10 m step length was best predicted by 3RM relative strength, while 0–5 m flight time was best predicted by 3RM absolute strength. The strength of these relationships was also low.

## Discussion

This study investigated the interactions between leg muscle qualities (power, stiffness, and strength), as measured by performance tests, with sprint acceleration velocity and step kinematics. As hypothesized, there were positive correlations between the leg power tests and velocity. The CMJ height (but not the calculated power index) related to and predicted velocity in all intervals, the relative 5BT and the RSI correlated with 5–10 m velocity, while the 5BT correlated with the 5–10 m and 0–10 m velocity (Tables 2 and 3). These results reinforce previous research that found leg power measured by jump test performance related to sprinting speed in athletes (Cronin and Hansen, 2005; Lockie et al., 2011; Nimphius et al., 2010; Peterson et al., 2006). However, the strength of the correlations was generally moderate, and the CMJ height predictive relationships were low. Additionally, there were no significant relationships found between leg power and step kinematics (Tables 2 and 3). There may be several reasons for this. The study sample drew from a relatively homogenous group of field sport athletes, which can limit the strength of correlations (Hopkins, 2000). Perhaps more importantly, these findings emphasize the complexity of sprint technique, where there is an interaction between many different physical and technical components (Hunter et al., 2004; Maulder et al., 2008). Leg power contributes to acceleration, but other physical factors are also required.

While it has been suggested that field sport athletes with faster acceleration may exhibit greater musculotendinous stiffness (Murphy et al., 2003), there were no significant relationships between sprint kinematics and leg stiffness within this study (Table 2). This supports Lockie et al. (2011), who found that bilateral or unilateral stiffness defined by hopping tests did not differentiate between faster and slower field sport athletes. Musculotendinous stiffness of the lower limb tends to increase with increased running velocity (Arampatzis et al., 1999), as a function of the need to attenuate greater ground contact impacts (Mero and Komi, 1987). Leg stiffness may be a more important factor in the maximum velocity phase of sprinting, as opposed to acceleration. In particular, leg stiffness may be influential in the 30–60 m zone of a maximal sprint (Bret et al., 2002).

Due to the need to shift the body mass during acceleration from a stationary or near-stationary position, relative strength is perhaps more important than absolute strength to sprint acceleration (Baker and Nance, 1999; Peterson et al., 2006). Although leg strength did not relate to sprint velocity, significant relationships were found between absolute and relative strength and 0–5 m flight time (Tables 2 and 3). The correlation suggested that greater strength related to a lower flight time, which had been recommended for field sport acceleration (Sayers, 2000). Greater relative strength also related to a longer 0–10 m step length, and improvements in leg strength had been shown to contribute to step length increases in a 10 m sprint (Lockie et al., 2012a). Potentially, stronger athletes can lengthen the step while still not spending an inordinate time in flight, which is pertinent for contact sports (the rugby codes, Australian and American football), as it can affect how athletes prepare themselves for contact with an opponent (Sayers, 2000). However, as for leg power, the strength of the correlations and predictive relationships were relatively low. This again highlights the complex interaction between physical factors important for sprinting speed and the technique produced, as well as the possible homogeneity of the sample.

There were limitations associated with this study. Correlation analyses do not prove cause-and-effect, and statistical models may be affected by factors not measured in a study (Brughelli et al., 2008). For example, stance kinetics were not measured, and the forces generated during stance can affect step kinematics produced by athletes (Lockie et al., 2013). The method by which leg stiffness was measured in this study (hopping at a set frequency of 2.2 Hz), which incorporated stiffness of the hip, knee and ankle joints by modelling the leg as a spring-mass system (Farley et al., 1991; Ferris and Farley, 1997), could have influenced the results. Future research should use methods that isolate leg joint stiffness, such as at the ankle (Spurrs et al., 2003) and knee (Toumi et al., 2004) joint, as this could provide different relationships with sprint acceleration. Leg strength was only measured bilaterally, and unilateral strength can influence linear speed (Lockie et al., 2012b). Beyond the 5BT, unilateral power was not assessed, and unilateral power had been linked to sprint performance (Lockie et al., 2014; McCurdy et al., 2010; Meylan et al., 2009). Forthcoming studies should further investigate the impact that unilateral strength and power has upon acceleration velocity and step kinematics in field sport athletes. Lastly, this study combined athletes from different sports. Specific athletic populations (e.g. soccer and rugby players) could be analyzed to ascertain whether they have a different interaction between leg muscle qualities and sprint acceleration.

In conclusion, within the context of these limitations, the results from this study indicate that there are selected relationships between 10 m sprint velocity and step kinematics, and leg muscle performance. The strength of the significant relationships highlighted the complexity of the interaction between leg muscle performance and sprint acceleration technique. Nevertheless, given the established relationships between leg power and strength with sprinting speed (Baker and Nance, 1999; Cronin and Hansen, 2005; Lockie et al., 2011; Nimphius et al., 2010), strength and conditioning coaches should still ensure their athletes develop leg muscle qualities that can manifest during acceleration.

## Figures and Tables

**Table 1:** Descriptive data (mean ± SD; 95% CI) for step length and frequency, and contact and flight time in the 0–5 m, 5–10 m, and 0–10 m intervals of a 10 m sprint in experienced male field sport athletes, and leg muscle performance tests (5BT, relative 5BT, CMJ height, CMJ power index, RSI, bilateral and unilateral leg stiffness [K], 3RM squat)

Variables	Mean (n = 28)	95% CI
0–5 m Step Length (m)	1.20 ± 0.11	1.15–1.24
5–10 m Step Length (m)	1.66 ± 0.11	1.61–1.70
0–10 m Step Length (m)	1.43 ± 0.10	1.39–1.47
0–5 m Step Frequency (Hz)	3.21 ± 0.26	3.11–3.31
5–10 m Step Frequency (Hz)	4.04 ± 0.31	3.92–4.16
0–10 m Step Frequency (Hz)	3.41 ± 0.23	3.31–3.49
0–5 m Contact Time (s)	0.148 ± 0.014	0.143–0.153
5–10 m Contact Time (s)	0.126 ± 0.010	0.122–0.130
0–10 m Contact Time (s)	0.140 ± 0.010	0.136–0.144
0–5 m Flight Time (s)	0.096 ± 0.014	0.090–0.101
5–10 m Flight Time (s)	0.125 ± 0.015	0.119–0.131
0–10 m Flight Time (s)	0.122 ± 0.012	0.107–0.117
5BT (m)	11.08 ± 0.95	10.71–11.45
Relative 5BT (m·kg^−1^)	0.135 ± 0.014	0.130–0.140
CMJ Height (m)	0.39 ± 0.05	0.37–0.41
CMJ Power Index (kg·m·s^−1^)	113.01 ± 13.74	107.78–118.43
RSI (CMJ height·s^−1^)	1.25 ± 0.26	1.15–1.35
Bilateral K (N·m·kg^−1^)	280.72 ± 24.41	271.25–290.18
Left-Leg K (N·m·kg^−1^)	226.97 ± 24.27	217.55–236.38
Right-Leg K (N·m·kg^−1^)	226.70 ± 31.03	214.67–238.73
3RM squat (kg)	119.09 ± 20.55	111.12–127.06
Relative 3RM Squat (kg·BM^−1^)	1.44 ± 0.20	1.36–1.52

**Table 2:** Correlations between sprint velocity (Vel) and step kinematics (step length [SL] and frequency [SF], contact [CT] and flight time [FT]) in the 0–5 m, 5–10 m, and 0–10 m intervals of a 10 m sprint, and leg power (5BT, relative 5BT [R5BT], CMJ height [CMJH], CMJ power index [CMJP], RSI); bilateral (Bi), left (L), and right (R) leg stiffness (K); and absolute and relative (R3RM) strength as measured by a 3RM squat

	5BT	R5BT	CMJH	CMJP	RSI	Bi K	L K	R K	3RM	R3RM
*0–5 m Interval*										
Vel	0.33	0.18	0.40*	0.31	0.20	−0.05	−0.22	−0.05	−0.02	−0.11
SL	0.25	0.16	0.16	0.13	−0.11	−0.19	0.11	0.19	−0.27	−0.37
SF	−0.04	−0.07	0.11	0.09	0.23	0.14	−0.21	−0.20	0.29	0.33
CT	−0.01	−0.13	−0.18	−0.02	−0.23	0.03	−0.02	0.07	−0.06	−0.16
FT	−0.14	0.10	0.17	−0.10	−0.02	−0.17	0.13	0.18	−0.51*	−0.46*
*5–10 m Interval*										
Vel	0.47*	0.43*	0.54*	0.26	0.37*	−0.03	−0.11	0.08	−0.22	−0.22
SL	0.02	0.04	0.21	0.09	−0.04	−0.16	0.07	0.13	−0.29	−0.33
SF	0.22	0.18	0.10	0.06	0.24	0.11	−0.14	−0.09	0.16	0.19
CT	−0.12	−0.13	−0.05	−0.03	−0.25	−0.15	−0.07	−0.06	−0.20	−0.24
FT	−0.26	−0.28	0.03	0.04	−0.17	−0.19	0.05	0.07	−0.07	−0.13
*0–10 m Interval*										
Vel	0.39*	0.28	0.47*	0.30	0.25	−0.07	−0.15	0.07	−0.10	−0.16
SL	0.14	0.09	0.20	0.12	−0.09	−0.19	0.09	0.17	−0.30	0.38*
SF	0.13	0.09	0.14	0.10	0.28	0.13	−0.21	−014	0.24	0.28
CT	−0.08	−0.12	−0.15	−0.04	−0.22	−0.05	−0.06	−0.03	−0.11	−0.17
FT	−0.27	−0.14	0.11	−0.04	−0.14	−0.24	0.16	0.19	−0.34	−0.34

* Significant (p ≤ 0.05) relationship between variables

**Table 3:** Stepwise linear regression analysis between 0–5 m, 5–10 m, and 0–10 m sprint velocity, and selected step kinematics that demonstrated significant predictive relationships, with leg power (5BT, relative 5BT, CMJ height, CMJ power index, and RSI), bilateral and unilateral absolute and relative leg stiffness, and absolute and relative leg strength as measured by a 3RM squat

Best Predictors of the Kinematic Variable	r	r^2^	Significance
0–5 m Velocity			
CMJ Height	0.40	0.16	0.04
5–10 m Velocity			
CMJ Height	0.54	0.29	<0.01
0–10 m Velocity			
CMJ Height	0.47	0.22	0.01
0–10 m Step Length			
3RM Relative Strength	0.38	0.14	0.05
0–5 m Flight Time			
3RM Absolute Strength	0.51	0.26	0.01
